# Development and Ex Vivo Evaluation of a Thermoreversible Silver Nanoparticle-Loaded Gel as a Biocompatible Intracanal Medicament

**DOI:** 10.3390/jfb17040180

**Published:** 2026-04-06

**Authors:** Shih-Min Hsia, Ming-Gene Tu, Wen-Hao Yang, Tong-Hong Wang, Yin-Hwa Shih, Tzong-Ming Shieh

**Affiliations:** 1 School of Nutrition and Health Sciences, College of Nutrition, Taipei Medical University, Taipei 110301, Taiwan; bryanhsia@tmu.edu.tw; 2 Nutrition Research Center, Taipei Medical University Hospital, Taipei 110301, Taiwan; 3 School of Dentistry, China Medical University, Taichung 404328, Taiwan; mgtu@mail.cmu.edu.tw (M.-G.T.); 112008801@cmu.edu.tw (W.-H.Y.); 4 Department of Dentistry, China Medical University Hospital, Taichung 404327, Taiwan; 5 BioBank, Chang Gung Memorial Hospital at Linkou, Taoyuan 333423, Taiwan; cellww@adm.cgmh.org.tw; 6 Department of Healthcare Administration, Asia University, Taichung 413305, Taiwan; 7 Institute of Oral Biology, College of Dentistry, National Yang Ming Chiao Tung University, Taipei 112304, Taiwan

**Keywords:** biofilm removal, cytotoxicity, endodontic disinfection, intracanal medicament, silver nanoparticles, thermoreversible gel

## Abstract

Inspired by their biocompatibility and thermoreversible gelation—transitioning from room temperature liquids to body temperature gels—Pluronic hydrogels were employed in this study to optimize intracanal penetration and ensure medicament stability. We developed a silver nanoparticle (AgNP)-loaded Pluronic gel (AgNPs-P-gel) as a biocompatible, easily removable intracanal medicament. Following PRILE 2021 guidelines, AgNPs-P-gels (F127/F68) were evaluated for gelation, AgNP release, and antibacterial activity against *Enterococcus faecalis* and *Streptococcus mutans* via minimum inhibitory concentration (MIC), minimum bactericidal concentration (MBC), and growth curves. Biofilms in bovine teeth were quantified using CFUs and scanning electron microscope (SEM) imaging. Biocompatibility was tested in L-929 fibroblasts using MTT assays and RT-qPCR for pro-inflammatory cytokines (*IL-6*, *TNF-α*, *IL-1β*). Removal efficacy from bovine canals was microscopically scored. The optimized formulation (20% F127, 7.5% F68) gelled at 34 °C with sustained release over 168 h. AgNPs-P-gel showed strong antibacterial activity (MIC: 25–50 µg/mL). In ex vivo models, 100 µg/mL AgNPs-P-gel (AgNPs-100-P-gel) reduced bacterial counts comparably to calcium hydroxide and chlorhexidine, but with lower cytotoxicity. Although inducing cytokine expression similar to conventional medicaments, AgNPs-P-gel demonstrated significantly superior removability. Thermoreversible AgNPs-P-gel offers sustained antimicrobial action, favorable biocompatibility, and superior removability, potentially improving endodontic disinfection predictability as a calcium hydroxide alternative.

## 1. Introduction

Successful endodontic treatment depends on a synergic combination of effective chemo-mechanical preparation, thorough irrigation, the strategic use of intracanal medicaments, and three-dimensional obturation. Although mechanical instrumentation and chemical irrigation are fundamental for bacterial reduction, they often fail to completely eliminate microorganisms due to the intricate anatomical complexity of the root canal system—such as isthmuses, lateral canals, and apical ramifications—and the development of microbial resistance [1,2]. Consequently, intracanal medicaments play a critical role in reducing the residual bacterial load and preventing reinfection between clinical appointments [3].

Persistent endodontic infections are frequently driven by resilient microorganisms, most notably *Enterococcus faecalis*, which is isolated in up to 32% of primary cases and nearly 90% of failed root canal treatments [4,5,6,7,8]. Unlike the Gram-negative anaerobes dominant in primary infections, *E. faecalis* thrives in secondary infections due to its remarkable ability to endure nutrient starvation and adhere strongly to dentin via specialized proteins, such as serine protease and collagen-binding protein [9,10]. Furthermore, its capacity for complex biofilm formation and intermediate resistance to multiple antibiotics—including gentamicin and metronidazole—significantly hinder conventional disinfection protocols [11,12,13]. In addition to *E. faecalis*, *Streptococcus mutans* serves as a primary cariogenic agent that can engage in complex microbial interactions within biofilms [14,15,16]. Its pathogenicity is driven by a robust metabolic capacity to synthesize organic acids and an inherent resilience to environmental stressors. Clinically, *S. mutans* is frequently isolated from infected root canals, and its presence is highly correlated with the pathogenesis of both acute apical abscesses and chronic endodontic infections, representing initial coronal biofilm or secondary contamination [17,18].

Currently, calcium hydroxide Ca(OH)_2_ remains the gold standard for intracanal medication due to its excellent biocompatibility and alkaline pH (11–12), which creates a lethal environment for most bacteria through the release of hydroxyl ions [19,20]. However, its antimicrobial action is often incomplete; *E. faecalis* can withstand the high alkalinity of Ca(OH)_2_ for over 10 days by utilizing sophisticated pH homeostasis mechanisms [21,22,23,24]. Furthermore, the thorough removal of Ca(OH)_2_ from irregular canal walls is technically challenging; its remnants can compromise the bonding strength and sealing ability of root canal sealers, ultimately jeopardizing the final obturation [25]. While alternative calcium hydroxide-based pastes containing iodoform, such as Metapex or Vitapex, offer synergistic antimicrobial properties, they are similarly difficult to remove completely once their therapeutic phase is complete [26]. Alternatively, 2% chlorhexidine (CHX) is effective against pathogens like *E. faecalis* and *Candida albicans*, but concerns persist regarding its concentration-dependent cytotoxicity and potential for triggering persistent inflammatory responses in connective tissues [27,28,29,30]. Furthermore, the interaction between CHX and sodium hypochlorite (NaOCl) can form a precipitate associated with parachloroaniline (PCA), which is linked to potential carcinogenicity and clinical tooth discoloration [31,32]. Therefore, an ideal intracanal medicament should combine antimicrobial activity, biocompatibility, sustained release, and easy removal—a combination not currently achieved by conventional medications.

Given these limitations, silver nanoparticles (AgNPs) have emerged as a promising tool in endodontics due to their potent, broad-spectrum antimicrobial efficacy against Gram-positive and Gram-negative pathogens, including multi-drug-resistant strains [33,34,35,36]. The bactericidal mechanism of AgNPs is primarily driven by oxidative dissolution and the subsequent liberation of silver ions, which bind to the bacterial cell wall and cytoplasmic membrane. This interaction results in heightened membrane permeability and irreversible structural deterioration [37,38,39,40,41]. AgNPs also exhibit anti-inflammatory effects and favorable biocompatibility, supporting their use across various endodontic applications [42,43,44]. However, several constraints still hinder the clinical application of AgNPs, particularly their high surface energy leading to a tendency to aggregate, dose-dependent cytotoxicity risks, and the significant challenges in removal from traditional viscous carriers—all of which necessitate a more sophisticated delivery system.

Pluronic F127 and F68 are FDA-approved, non-toxic, thermoreversible polymers widely used in advanced drug delivery systems [45,46]. These biologically inert poloxamers transition from a liquid form at low temperatures to a stable gel at body temperature, allowing for ease of injection and optimal retention within the complex canal anatomy [47]. The gelation temperature can be modulated by adjusting the polymer concentration to suit specific clinical needs [48]. Integrating AgNPs into such a thermoreversible hydrogel (P-gel) represents a promising strategy to overcome the limitations of AgNPs. This system ensures uniform dispersion of AgNPs in the liquid phase to minimize aggregation and provides a sustained release profile that reduces overall cytotoxicity by preventing sudden cellular exposure to high concentrations [49]. While existing studies have evaluated the cytotoxicity and antimicrobial efficacy of AgNP-loaded gels, there remains a significant lack of comprehensive assessment regarding drug removal and the resulting inflammatory response in a clinical context.

This study aims to develop a thermoreversible AgNPs-P-gel and evaluate its physicochemical and biological properties, including gelation temperature, flowability, antimicrobial efficacy, cytotoxicity, pro-inflammatory gene expression, and ease of removal. The AgNPs-P-gel was compared against Ca(OH)_2_ and 2% CHX, which are conventional intracanal medicaments. We hypothesized that a thermoreversible AgNP-loaded gel would provide sustained antimicrobial activity comparable to conventional medicaments while improving removability and reducing systemic cytotoxicity.

## 2. Materials and Methods

In clinical settings, biofilms are indeed complex and multispecies. However, the use of a standardized mono-species biofilm model was intentionally chosen for this study to isolate and precisely quantify the antimicrobial efficacy of the AgNPs-P-gel. The manuscript of this laboratory study has been written according to Preferred Reporting Items for Laboratory studies in Endodontology (PRILE) 2021 guidelines.

### 2.1. Bacterial Culture and OD600 Measurement

*Enterococcus faecalis* (ATCC 29212) and *Streptococcus mutans* (ATCC 25175) were used in this study. Optical density at 600 nm (OD600) was measured in a plastic cuvette using a spectrophotometer (GeneQuant 100, Harvard Bioscience, Holliston, MA, USA). Bacterial suspensions were adjusted to an OD600 of 1.0 (approximately 1 × 10^9^ CFU/mL). Each well received 200 μL of test medicament, 2 μL of bacterial suspension, and 48 μL of TSB, yielding a final volume of 250 μL. After incubation at 37 °C for 24 h, OD600 was measured again.

### 2.2. Minimum Inhibitory Concentration (MIC) and Minimum Bactericidal Concentration (MBC)

MIC and MBC of silver nanoparticles (AgNPs) (Product Code: DA02; Liwei Nano Tech. Co., Ltd., Kaohsiung, Taiwan) were determined by broth microdilution in TSB using two-fold serial dilutions (3.125–100 µg/mL). *E. faecalis* suspensions (OD600 = 1.0) were incubated in 96-well plates at 37 °C for 24 h. MIC was the lowest concentration with OD600 comparable to the background. MBC was determined by spot-plating onto TSB agar (Neogen, Lansing, MI, USA) and defined as ≥99% reduction in viable counts. Assays were triplicated [50].

### 2.3. Preparation and Characterization of Thermoreversible AgNPs-P-gel

The thermoreversible AgNPs-P-gel was prepared using the “cold method”. The polymer matrix consisted of Pluronic F127 (Kolliphor^®^ P407 Micro) and Pluronic F68 (Kolliphor^®^ P188 Micro), both sourced from Wei Ming Pharmaceutical Co., Ltd. (Taipei, Taiwan). AgNPs were obtained as a 2000 ppm aqueous stock dispersion (Product Code: DA02; Liwei Nano Tech. Co., Ltd., Kaohsiung, Taiwan). According to the manufacturer, the AgNPs are spherical with an average particle size of 15 nm and are stabilized in aqueous dispersion. All raw materials were stored at 4 °C in the dark prior to use to maintain stability and prevent AgNP aggregation. To prepare the formulations, specific concentrations of AgNP dispersions (20, 50, and 100 ppm) were pre-cooled to 4 °C. Pluronic F127 (400 mg) and F68 (150 mg) were added to 2000 μL of the chilled AgNP dispersion or sterile double-distilled water (for the Water-P-gel control). The mixtures were vortexed thoroughly until a clear, homogenous solution was formed. All tubes and pipette tips were pre-cooled to 4 °C to prevent premature gelation during handling.

The experimental groups were defined as follows:

- TSB group: Tryptic soy broth (TSB; BD Life Sciences, East Rutherford, NJ, USA) only (negative control).
- Water-P-gel group: Pluronic matrix dissolved in sterile double-distilled water.
- AgNPs-20/50/100-P-gel groups: Pluronic matrix dissolved in 20, 50, or 100 ppm AgNP dispersions, respectively.
- 2% CHX group: 2% chlorhexidine gluconate (CHX; C9394, Sigma—Aldrich, Burlington, MA, USA) (positive control).

### 2.4. Determination of Gelation Temperature

The gelation temperature was determined using the tube inversion method [49]. Samples (2 mL) were placed in glass vials and submerged in a temperature-controlled water bath. The temperature was increased from 20 °C to 35 °C at a constant rate of 1 °C every 5 min. At each degree increment, the vials were tilted to 45 °C and 90 °C. Gelation temperature was defined as the temperature at which no fluid flow was observed within 30 s of inversion. Formulations exhibiting a rapid sol–gel transition between 25 ± 2 °C (fluid state) and 33 ± 2 °C (gel state) were selected for further characterization.

### 2.5. Flowability Analysis

The flowability of the experimental hydrogels was evaluated following the ISO 6876:2001 standard for dental root canal sealing materials. To account for the thermoreversible nature of the Pluronic-based system, all testing components—including glass plates (40 × 40 × 5 mm), delivery tips, and the hydrogel samples—were pre-conditioned at three specific temperatures: 4 °C, 20 °C (room temperature), and 37 °C (physiological temperature). Briefly, 50 μL of the well-mixed hydrogel (water-P-gel or AgNPs-100-P-gel) was dispensed onto the center of a glass plate. After a 3 min stabilization period at the respective temperature, a second glass plate was placed over the sample, followed by the application of a 100 g weight to compress the material. After 10 min of loading, the weight was removed, and the maximum and minimum diameters of the compressed gel were measured using a digital caliper. In accordance with ISO 6876:2001, the flowability was recorded as the mean of the two diameters, provided the difference between them was less than 1 mm. To further evaluate the material’s response to pressure, additional tests were conducted using a reduced load of 10 g. All measurements were performed in triplicate.

### 2.6. Bacterial Growth Curve

Bacterial growth curves were monitored using a Versa Max Microplate reader (Avantor, Radnor, PA, USA) by recording OD600 at 2 h intervals from 2 to 24 h. All experiments were conducted in triplicate. Wells containing only TSB served as untreated controls. The antibacterial effect of each medicament was evaluated by comparing OD600 values over time [51].

### 2.7. Bovine Tooth Preparation

The experimental bovine teeth were purchased from Homo Enterprise Co., Ltd. (Pingtung, Taiwan). The animal research protocol has been approved (CMUIACUC-2025-304). Non-carious, single-rooted bovine incisors (20 mm length) were instrumented to #80 K-file (size: 15/40, length: 25 mm, lot number: 031156445, SybronEndo, Glendora, CA, USA) using a step-back technique, irrigated with 3% NaOCl (Ref 40019, Prevest DenPro, Jammu, India) between files, and finally rinsed with 5 mL 17% EDTA (Well-Prep, Vericom, Gangwon-do, Korea), 5 mL 3% NaOCl, and 5 mL distilled water. Apical foramina were sealed with nail varnish, and specimens were autoclaved at 121 °C for 30 min before use.

### 2.8. Biofilm Model Using Bovine Teeth

*Enterococcus faecalis* suspensions (OD600 = 1.0) were used to inoculate prepared bovine root canals. Based on prior findings [52], immature biofilms were formed after 2 days, and mature biofilms after 4 days (TSB refreshed at 48 h). Pre-medication bacterial counts (CFUs/mL) were recorded, medicaments were applied to the canal orifice, and samples were incubated (immature: 24 h; mature: 7 days). Post-treatment, canals were irrigated with 5 mL sterile saline and CFUs/mL were re-measured. Six subgroups (*n* = 5) were tested: TSB control, water-P-gel, AgNPs-50-P-gel, AgNPs-100-P-gel, Ca(OH)_2_ paste (Well-Pex, WP102-K000 Vericom, Gangwon-do, Korea), and 2% chlorhexidine gluconate (CHX; C9394, Sigma—Aldrich, Burlington, MA, USA). Sterility controls (*n* = 3) contained only sterile TSB.

#### 2.8.1. Biofilm Cultivation and Standardization

The primary experimental pathogen, *Enterococcus faecalis*, was cultured in TSB under aerobic conditions at 37 °C for 24 h. To ensure baseline consistency across all samples, the bacterial density was standardized to an optical density of OD600 = 1 using a spectrophotometer. Building upon established evidence that *E. faecalis* monospecies biofilms achieve structural maturation after approximately 72 h [52], the experimental framework was categorized into two distinct models: an immature biofilm model (short-term treatment) and a mature biofilm model (long-term treatment).

#### 2.8.2. Placement of Intracanal Medicament

The teeth were randomly assigned to 6 subgroups (5 samples each).

- TSB group: treated with TSB.
- Water-P-gel group: treated with water-based gel (400 μg F127 and 150 μg F68 dissolved in 2000 μL sterile double-distilled water).
- AgNPs-50-P-gel group: treated with 20 μg/mL AgNPs-Pluronic gel (400 μg F127 and 150 μg F68 dissolved in 2000 μL 50 ppm AgNPs solution).
- AgNPs-50-P-gel group: treated with 50 μg/mL AgNPs-Pluronic gel (400 μg F127 and 150 μg F68 dissolved in 2000 μL 50 ppm AgNPs solution).
- AgNPs-100-P-gel group: treated with 100 μg/mL AgNPs-Pluronic gel (400 μg F127 and 150 μg F68 dissolved in 2000 μL 100 ppm AgNPs solution).
- Ca(OH)_2_ group: treated with Ca(OH)_2_ paste.
- 2% CHX: treated with 2% CHX.

Moreover, three samples were filled only with TSB without any bacteria. These three specimens were used for sterility control.

#### 2.8.3. Immature Biofilm Model (24 h Treatment, *n* = 30)

For the immature model, individual bovine teeth were secured in Eppendorf tubes. The root canals were inoculated with the standardized *E. faecalis* suspension until the fluid reached the canal orifice. To facilitate initial biofilm attachment and development, the samples were incubated undisturbed at 37 °C for 48 h. Following this incubation, baseline microbial loads were established by quantifying pre-medication colony-forming units, with the data converted to a logarithmic scale and expressed as pre-log CFUs/mL. The assigned intracanal medicaments were then delivered into the canals until visible at the orifice. After a precise 24 h exposure period, each canal was meticulously irrigated with 5 mL of sterile saline to remove non-adherent bacteria. Post-medication microbial levels were then quantified and similarly converted to post-log CFUs/mL. To evaluate the immediate antimicrobial potency of the agents, the efficacy for each bovine tooth was assessed by calculating the log reduction, determined by subtracting the pre-log CFUs/mL from the post-log CFUs/mL. Statistical comparisons between the different treatment groups were then performed based on the mean values of these log CFU/mL changes (log reduction) measured before and after medicament application.

log CFUs/mL Reduction = (pre-log CFUs/mL) − (post-log CFUs/mL)

#### 2.8.4. Mature Biofilm Model (7-Day Treatment, *n* = 30)

The mature model followed a more rigorous cultivation protocol to simulate chronic infection. After initial inoculation, the samples underwent a 4-day maturation phase. To maintain bacterial viability and encourage the development of a complex biofilm matrix, the TSB growth medium was refreshed after the first 48 h. Once maturation was complete, baseline microbial levels were established by measuring pre-medication colony-forming units, which were then converted to a logarithmic scale and recorded as pre-log CFUs/mL. The assigned intracanal medicaments were then delivered into the canals and maintained within the root canal system for a sustained duration of 7 days. This prolonged exposure period was specifically designed to evaluate the persistence and penetrative capacity of the medicaments against established microbial communities. Following the 7-day treatment, each canal was rinsed with 5 mL of sterile saline. Post-medication microbial levels were then quantified and converted to post-log CFUs/mL. To determine the definitive antibacterial effectiveness against these established biofilms, the log reduction for each bovine tooth was calculated by subtracting the pre-log CFUs/mL from the post-log CFUs/mL. Statistical evaluations were conducted by comparing the mean values of these log-CFU/mL changes across the different treatment groups.

### 2.9. Bacterial Quantification

To evaluate the antimicrobial efficacy of the various medicaments, the microbial concentration within the root canals was quantified before and after treatment using the colony-forming unit (CFU/mL) counting method [49]. Sterile paper points (Meta-Biomed, Chungcheongbuk-do, Republic of Korea) were inserted into each root canal and maintained for 1 min to absorb the intracanal fluid and associated bacteria. Each paper point was then immediately transferred into a microcentrifuge tube containing 1 mL of TSB. To ensure that the bacteria were completely dislodged from the paper fibers and into the liquid medium, the tubes underwent vigorous vortexing for 1 min. The resulting bacterial suspension was homogenized to ensure a uniform distribution of cells. To achieve a countable number of colonies on the agar surface, serial dilutions were performed as follows:

Pre-medication evaluation: Given the high initial bacterial load, the suspension was diluted to a factor of 10,000-fold.

Post-medication evaluation: To account for the expected reduction in bacterial viability following treatment, a 1000-fold dilution was utilized.

A precise aliquot of 100 μL from each standardized dilution was spread evenly across the surface of TSB agar plates using a sterile spreader. The plates were then placed in a bacteriological incubator at 37 °C for 24 h under aerobic conditions. Following the incubation period, plates with distinct, non-overlapping colonies were selected for counting. The final microbial concentration was calculated using the following formula:

CFU/mL = (Number of colonies × Dilution factor)/Volume of inoculum (mL)

This quantitative approach allowed for a precise comparison of the bacterial reduction achieved by the AgNPs-Pluronic gel relative to traditional intracanal medicaments [53,54,55].

### 2.10. Scanning Electron Microscopy (SEM) Analysis

Bovine incisors were sectioned, irrigated with 3% NaOCl and 17% EDTA, autoclaved, and inoculated with *E. faecalis* (OD600 = 1) for 3 days at 37 °C (TSB refreshed on day 2). After PBS rinsing, specimens received assigned medicaments for 48 h and then were rinsed again. Groups: untreated, biofilm only, PBS, AgNPs-100-P-Gel, and 2% CHX.

The samples were divided into 5 groups:

- Untreated Group: The teeth were left completely untreated, without bacterial inoculation or medicament placement, confirming the process of sterilization as well as the smear layer removal.
- Non-Medicated Group (biofilm only): After biofilm maturation, no medicament was applied, allowing observation of the biofilm morphology.
- PBS Group: Following biofilm maturation, the teeth were immersed in PBS.
- AgNPs-100-P-gel Group: Following biofilm maturation, the teeth were immersed in 100 μg/mL AgNPs-Pluronic gel.
- 2% CHX Group: Following biofilm maturation, the teeth were immersed in 2% CHX.

The cells were fixed using 4% glutaraldehyde solution for 2 h, dehydrated in a series of ethanol washes, and dried with tertiary butyl alcohol in a vacuum oven (VOS-301SD, Eyela, Tokyo, Japan). Specimens were sputter-coated with gold–palladium in an ion coater (IB-3, ion coater, Eiko, Tokyo, Japan), and the root canal surfaces were observed using the Zeiss UltraPlus (Jena, Germany) SEM to assess morphology and bacterial presence at National Chung Hsing University. SEM was operated at 3.0 kV and a working distance of 5.5–12 mm under ×2000–×4000 magnifications to assess morphology and bacterial presence.

### 2.11. AgNP Release from AgNPs-100-P-Gel

To assess the release profile of AgNPs from the AgNPs-100-P-gel, 100 µL of the gel was transferred into a sterile Eppendorf tube and incubated at 37 °C for 30 min to simulate intracanal conditions. Subsequently, 1000 µL of PBS was added to the tube to initiate the release process. Based on the absorption spectrum of AgNPs, the characteristic peak for the AgNP suspension was identified at 428 nm. Therefore, at predetermined time points (1, 6, 12, 24, 48, 72, 120, 144, and 168 h), 500 µL of the release medium was collected, and its absorbance at 428 nm was measured using a Varioskan LUX Multimode Microplate Reader (Thermo Fischer Scientific, Waltham, MA, USA) to quantify the amount of AgNPs released. All measurements were performed in triplicate.

### 2.12. Cell Culture

L-929 mouse fibroblasts (BCRC RM60091) were used to evaluate the cytotoxicity of the test medicaments. Cells were maintained in Dulbecco’s Modified Eagle Medium (DMEM; Thermo Fisher Scientific, Waltham, MA, USA) supplemented with 10% fetal bovine serum (FBS; Thermo Fisher Scientific, Waltham, MA, USA), 5 mM L-glutamine (Thermo Fisher Scientific, Waltham, MA, USA), and 1% antibiotics (Thermo Fisher Scientific, Waltham, MA, USA) and incubated in a humidified atmosphere at 37 °C with 5% CO_2_. Adherent cells were passaged three times per week and detached using trypsin prior to seeding.

### 2.13. Cell Viability 

Extracts were prepared by incubating 1 g of each material in 5 mL DMEM at 37 °C for 24 or 72 h and were centrifuged, and supernatants were collected. L-929 cells (1 × 10^5^/mL) were seeded (100 µL/well) in 96-well plates, incubated for 24 h, and then exposed to extracts or fresh DMEM (control) for 24 or 72 h. MTT solution (1 mg/mL) (Thermo Fisher Scientific, Waltham, MA, USA) was added for 4 h and was replaced with 50 µL DMSO to dissolve formazan, and absorbance at 590 nm was measured to determine viability [51,56].

### 2.14. Pro-Inflammatory Cytokine Gene Expression

RT-qPCR was used to assess L-929 fibroblast inflammatory gene expression. Cells (1 × 10^5^) were seeded in 6-well plates, cultured for 24 h, and then exposed to DMEM or test extracts for 24 h. Total RNA was extracted with TRI reagent (Thermo Fisher Scientific, Waltham, MA, USA), reverse-transcribed using Oligo(dT)_20_ primers, and analyzed by quantitative PCR (qPCR) for *interleukin-1β (IL-1β)*, *tumor necrosis factor-α (TNF-α)*, and *interleukin-6 (IL-6)*, normalized to *glyceraldehyde 3-phosphate dehydrogenase (GAPDH)*. The qPCR was performed by a StepOnePlus Real-Time PCR System and PowerUp SYBR Green Master Mix (Applied Biosystems, Waltham, MA, USA). Primer sequences are listed in Table 1. All experiments were triplicated, and data were processed per established protocols [51].

### 2.15. Removal Efficacy

Sixteen bovine incisors were decoronated to 20 mm, instrumented to size #80 K-file with the step-back technique, and irrigated with 3% NaOCl, 17% EDTA, 3% NaOCl, and distilled water. Apical foramina were sealed, and canals were dried and medicated with calcium hydroxide paste or AgNPs-100-P-gel; four served as controls. After 7 days at 37 °C and 100% humidity, specimens were assigned to two removal methods. Residual medicament was scored by a single, experienced dentist to ensure a consistent interpretive baseline. To maintain high diagnostic reliability, an intra-examiner calibration procedure was performed before the formal evaluation, where a subset of samples was scored twice at a two-week interval, achieving an intra-examiner kappa coefficient of 0.87, indicating substantial agreement.

### 2.16. Standard Needle Irrigation (SNI)

Specimens were irrigated with 5 mL 3% NaOCl for 1 min and then 5 mL 17% EDTA for 1 min using a syringe positioned 1–2 mm short of the working length without contacting canal walls. Canals were rinsed with saline, and roots were split longitudinally and imaged.

### 2.17. Hand File Irrigation (HFI)

The master apical file was gently moved to working length for 1 min while irrigating with 5 mL 3% NaOCl. Canals were dried, split, and photographed. Then, 17% EDTA was applied to the canal walls for 3 min, rinsed with 5 mL saline, and imaged again.

### 2.18. Statistical Analysis

OD600, MIC, biofilm CFU reduction, cell viability, pro-inflammatory cytokine gene expression, and AgNP release were analyzed by one-way ANOVA, followed by Tukey’s post hoc test when applicable. Remnant scores were compared using an unpaired *t*-test. All data were pre-screened using the Shapiro–Wilk normality test. Significance was set at *p* ≤ 0.05. Analyses and graphs were generated with GraphPad Prism 10.2.3.

## 3. Results

### 3.1. MIC and MBC of AgNPs Against E. faecalis and S. mutans

**Figure 1:**
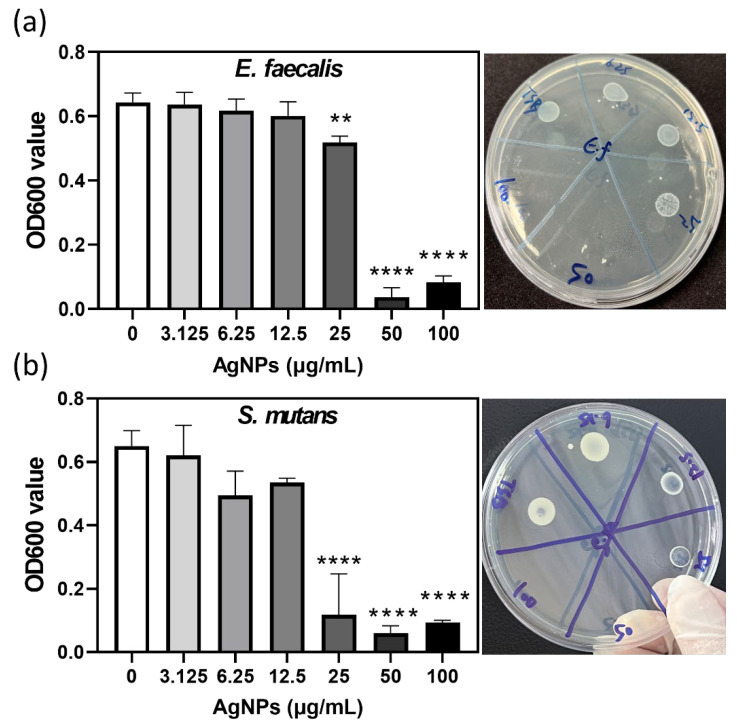
Antibacterial activity of AgNPs. MIC and MBC of AgNPs against *E. faecalis* and *S. mutans*. OD600 measurements for *E. faecalis* ((**a**), left) and *S. mutans* ((**b**), left) were obtained after exposure to 3.125–100 µg/mL AgNP concentrations. Spot-plating assays confirmed complete eradication of both species at 50 μg/mL ((**a**), right; (**b**), right). Data are mean ± SD. AgNP-treated groups were compared with the untreated control (0 μg/mL). ** *p* < 0.01, **** *p* < 0.0001; one-way ANOVA. Abbreviation: OD, optical density.

After 24 h aerobic incubation at 37 °C, *E. faecalis* showed turbidity at 3.125–25 µg/mL AgNPs, but not at 50 or 100 µg/mL. Agar plate inoculation confirmed bactericidal activity at 50 and 100 µg/mL, establishing both MIC and MBC at 50 µg/mL (Figure 1a). For *S. mutans*, turbidity occurred at 3.125–12.5 µg/mL, with complete inhibition at ≥25 µg/mL. Plate assays confirmed MBC at 50 µg/mL and MIC at 25 µg/mL (Figure 1b).

### 3.2. Gelation Temperature and Bacterial Growth in AgNPs-P-Gel

**Figure 2:**
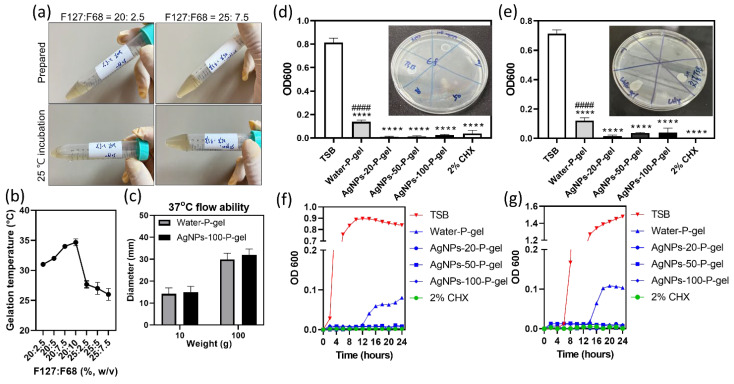
Gelation properties and antibacterial activity of AgNPs-P-gel. (**a**) Representative images showing the physical state of Pluronic formulations at room temperature (~20 °C) and at 25 °C. Formulations with F127:F68 ratios of 20:2.5 (left) and 25:7.5 (right) were liquid at room temperature. Upon heating to 25 °C, the 20:2.5 formulation remained liquid (bottom left), whereas the 25:7.5 formulation formed a gel (bottom right). (**b**) Gelation temperatures of different F127:F68 formulations. (**c**) Flowability of water-P-gel and AgNPs-100-P-gel. 100 g or a reduced 10 g load was applied. (**d**,**e**) OD600 measurements of *E. faecalis* (**d**) and *S. mutans* (**e**) after treatment with water-P-gel, AgNPs-20-P-gel, AgNPs-50-P-gel, AgNPs-100-P-gel, or 2% CHX. Corresponding spot-plating assays (insets, top right) confirmed complete bacterial eradication in AgNPs-P-gel and 2% CHX-treated groups. (**f**,**g**) Growth curves of *E. faecalis* (**f**) and *S. mutans* (**g**) under the same treatment conditions as in (**c**,**d**). Data are presented as mean ± standard deviation (SD). Statistical comparisons were performed between AgNPs-P-gel groups and the untreated control (TSB group) (**** *p* < 0.0001) and between AgNPs-P-gel groups and the positive control (2% CHX group) (#### *p* < 0.0001) using one-way ANOVA with Tukey’s post hoc test. Abbreviations: CHX, chlorhexidine; TSB, tryptic soy broth; OD, optical density.

Consistent with prior reports [57], 25% F127 P-gel had a lower gelation temperature than 20% F127. Adding F68 increased gelation temperature in 20% F127 gels but decreased it in 25% F127 gels. The 20% F127 + 7.5% F68 formulation (~34 °C) was selected for further preparation (Figure 2a,b). The flowability testing follows the ISO 6876:2001 standard. All components were pre-conditioned at 4 °C, 20 °C, and 37 °C. While the flowability at 4 °C and 20 °C exceeded the measurable limit (>40 mm), both water-P-gel and AgNPs-100-P-gel maintained a flow of approximately 30 mm at 37 °C. This outperforms the 26–27 mm previously recorded for Apexit Plus using the same method [58]. Our findings confirm that both experimental gels meet ISO requirements and that the addition of 100 ppm AgNPs does not statistically alter the material’s flow properties, even under varying loads (100 g vs. 10 g) (Figure 2c).

AgNPs-P-gels (20–100 µg/mL) fully inhibited bacterial growth, confirmed by OD600 readings and absence of colonies at 24 h, similar to 2% CHX (Figure 2d,e). In growth curves, *E. faecalis* and *S. mutans* showed rapid proliferation in TSB (OD600 = 0.9–1.5, growth onset 2–6 h), delayed growth in water-P-gel (onset ~12 h, OD600 < 0.1), and complete suppression with AgNPs-P-gels or 2% CHX (Figure 2f,g). The prolonged lag phase in water-P-gel suggests restricted mobility and nutrient access, yielding mild inhibition without bactericidal activity.

### 3.3. Antibacterial Effects of AgNPs-P-Gels Against Immature and Mature E. faecalis Biofilms in Bovine Teeth

**Figure 3:**
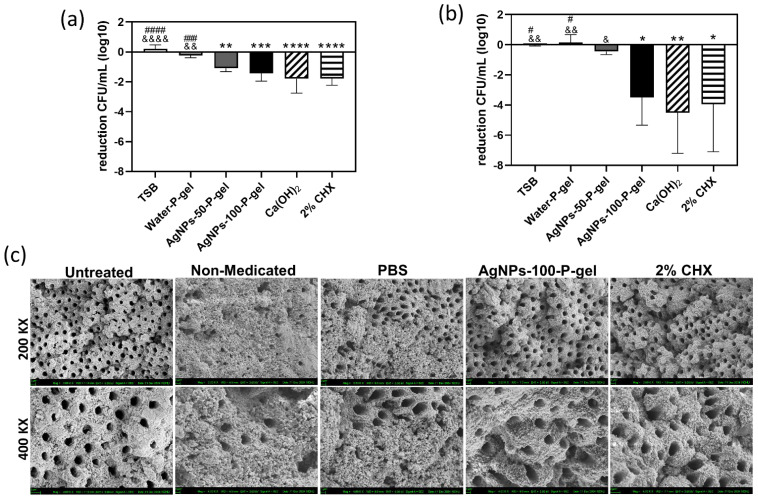
Antibacterial effects of AgNPs-P-gels against immature and mature *E. faecalis* biofilms in bovine root canals. Antimicrobial activity of water-P-gel, AgNPs-50-P-gel, AgNPs-100-P-gel, Ca(OH)_2_, and 2% CHX was evaluated in an ex vivo bovine tooth model. (**a**) Immature biofilm model: Biofilms were grown for 48 h, followed by 24 h of treatment. (**b**) Mature biofilm model: Biofilms were grown for 4 days, followed by 7 days of treatment. Bacterial viability was quantified and expressed as mean ± standard deviation (SD). Statistical analysis was performed using one-way ANOVA with Tukey’s post hoc test. Significance was indicated for comparisons with the untreated control (TSB): * *p* < 0.05, ** *p* < 0.01, *** *p* < 0.001, and **** *p* < 0.0001, and for comparisons of AgNPs-P-gel groups with positive controls: Ca(OH)_2_ (& *p* < 0.05, && *p* < 0.01, &&&& *p* < 0.0001), and 2% CHX (# *p* < 0.05, ### *p* < 0.001, #### *p* < 0.0001). (**c**) Scanning electron microscopy (SEM) of bovine root canal dentin after treatment with untreated control, PBS, AgNPs-100-P-gel, and 2% CHX. Upper images: 2000× magnification; lower images: 4000× magnification. Scale bar = 2 μm.

Bacterial susceptibility to antimicrobials varies significantly with growth status—whether in planktonic, immature, or mature biofilm forms. In this study, an in vitro bovine root canal model infected with *E. faecalis* was utilized. In the immature biofilm model (48 h formation, 24 h treatment), the log CFU/mL reduction (mean ± SD) for each group was as follows: TSB (0.2048 ± 0.2624), water-P-gel (−0.2420 ± 0.1464), AgNPs-50-P-gel (−1.098 ± 0.2291), AgNPs-100-P-gel (−1.433 ± 0.5274), Ca(OH)_2_ (−1.797 ± 0.9633), and 2% CHX (−1.774 ± 0.4571). Compared to the TSB control, all medicament treatments significantly reduced CFU counts, with the exception of the water-P-gel group, which showed no significant inhibitory effect. No significant differences were observed between the AgNPs-50-P-gel, AgNPs-100-P-gel, Ca(OH)_2_, and 2% CHX groups (Figure 3a). In the mature biofilm model (4 days formation, 7 days treatment), the log CFU/mL reduction (mean ± SD) was: TSB (−0.007063 ± 0.07975), water-P-gel (0.1445 ± 0.5290), AgNPs-50-P-gel (−0.4574 ± 0.1930), AgNPs-100-P-gel (−3.507 ± 1.831), Ca(OH)_2_ (−4.510 ± 2.698), and 2% CHX (−3.935 ± 3.163). While most treatments reduced CFU counts compared to the TSB control, the water-P-gel and AgNPs-50-P-gel groups failed to show a significant antibacterial effect. Notably, the antibacterial efficacy of AgNPs-50-P-gel was insufficient against mature biofilms. In contrast, the AgNPs-100-P-gel, Ca(OH)_2_, and 2% CHX groups all achieved a reduction of >3 log_10_ CFU/mL. According to the National Committee for Clinical Laboratory Standards (NCCLS) guidelines, an antimicrobial treatment achieves ≥3 log_10_ CFU/mL reduction, equivalent to a 99.9% reduction [59]. No significant differences were found among these three groups. The relatively large standard deviations observed in these groups were attributed to the variation in bovine samples, where some specimens showed complete eradication (zero colony growth) after treatment, while others retained residual colonies (Figure 3b). Overall, AgNPs-100-P-gel maintained robust antibacterial activity against both immature and mature biofilms, demonstrating efficacy comparable to the gold standards, calcium hydroxide and 2% CHX.

### 3.4. SEM Observations of Biofilm Removal on Root Canal Dentin

SEM imaging focused on the coronal root canal for sample consistency. The control group showed no microorganisms, confirming effective sterilization. In the non-medicated group, *E. faecalis* biofilm extensively covered the dentin and penetrated dentinal tubules. The PBS group showed reduced surface biofilm, but tubules remained blocked by angular deposits, likely dehydrated PBS salts and residual biofilm. In contrast, both AgNPs-100-P-gel and 2% CHX groups displayed clean dentin surfaces without *E. faecalis*, salt crystals, or treatment residues (Figure 3c), indicating effective biofilm removal and surface cleanliness.

### 3.5. Cell Viability of AgNPs-P-Gel

**Figure 4:**
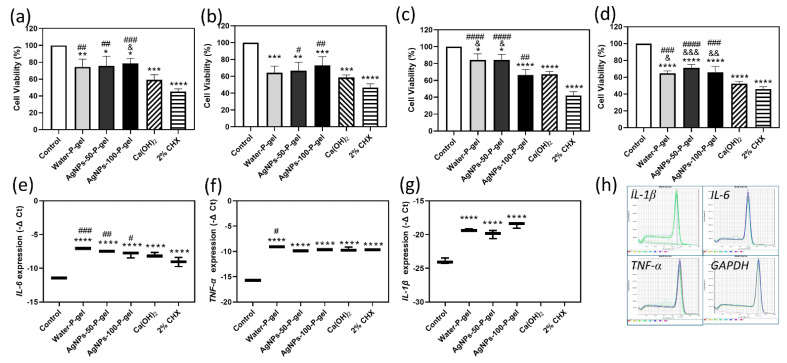
Cytotoxicity and pro-inflammatory cytokine gene expression induced by AgNPs-P-gel compared with calcium hydroxide and 2% CHX. Cytotoxic effects of different treatments on L-929 fibroblasts were assessed by the MTT assay under four extraction/exposure conditions: (**a**) 24 h extract + 24 h exposure; (**b**) 24 h extract + 72 h exposure; (**c**) 72 h extract + 24 h exposure; and (**d**) 72 h extract + 72 h exposure. Ca(OH)_2_ and 2% CHX were used as positive controls. Relative mRNA expression of *IL-6* (**e**), *TNF-α* (**f**), and *IL-1β* (**g**) was determined by RT-qPCR following 24 h exposure to medicament extracts; *IL-1β* transcripts were undetectable in the Ca(OH)_2_ and 2% CHX groups. (**h**) Melting curve analysis for *IL-6*, *TNF-α*, *IL-1β*, and *GAPDH* confirmed primer specificity. Data are expressed as mean ± SD. Statistical comparisons were performed between each treatment group (water-P-gel, AgNPs-P-gel, Ca(OH)_2_, 2% CHX) and the untreated control (DMEM): * *p* < 0.05, ** *p* < 0.01, *** *p* < 0.001, and **** *p* < 0.0001, and between AgNPs-P-gel and the positive controls Ca(OH)_2_ (& *p* < 0.05, && *p* < 0.01, &&& *p* < 0.001) or 2% CHX (# *p* < 0.05, ## *p* < 0.01, ### *p* < 0.001, #### *p* < 0.0001) using one-way ANOVA with Tukey’s post hoc test.

Across all conditions, 2% CHX showed the lowest cell viability, significantly lower than AgNPs-50-P-gel and AgNPs-100-P-gel after both 24 h extraction/24 h exposure and 24 h extraction/72 h exposure (Figure 4a,b). With 72 h extraction/24 h exposure, 2% CHX remained the lowest, followed by AgNPs-100-P-gel and calcium hydroxide, while water-P-gel and AgNPs-50-P-gel had the highest viability (Figure 4c). Under 72 h extraction/72 h exposure, 2% CHX and calcium hydroxide were lowest (Figure 4d). Overall, AgNPs-P-gels consistently showed higher viability, while 2% CHX was most cytotoxic.

### 3.6. Pro-inflammatory Cytokine Gene Expression

After 24 h extraction and 24 h exposure to L-929 cells, all medicaments increased *IL-6* (Figure 4e), *TNF-α* (Figure 4f), and *IL-1β* (Figure 4g) expression, except *IL-1β*, which was undetectable in the calcium hydroxide and 2% CHX groups. *GAPDH* and target genes showed single sharp melting peaks, confirming primer specificity (Figure 4h). Pluronic-based water-P-gel induced pro-inflammatory cytokines, in some cases exceeding 2% CHX.

### 3.7. AgNP Release of AgNPs-P-Gel

**Figure 5:**
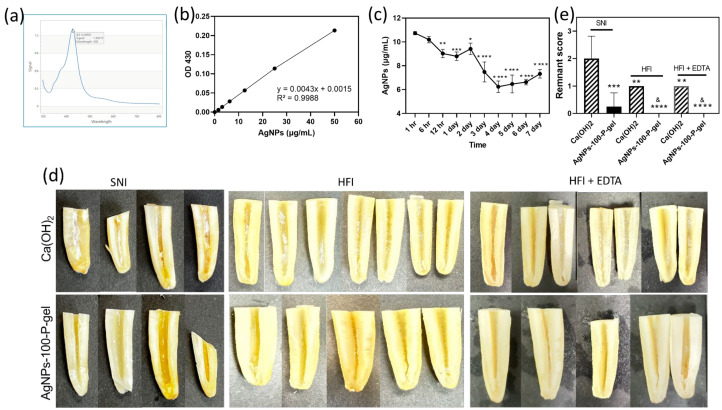
Release kinetics of AgNPs from AgNPs-P-gel and residual medicament assessment in root canals. (**a**) Full-wavelength spectrum of AgNPs, showing the maximal absorbance peak at ~428 nm. (**b**) Standard calibration curve generated from serial two-fold dilutions of AgNPs (50 μg/mL stock in PBS), with OD measured at 428 nm (R^2^ = 0.9988). (**c**) Cumulative release profile of AgNPs from AgNPs-100-P-gel over 7 days. Values are presented as mean ± standard deviation (SD). Statistical differences were determined relative to the 1 h time point: * (*p* < 0.05), ** (*p* < 0.01), *** (*p* < 0.001), **** (*p* < 0.0001), one-way ANOVA with Tukey’s post hoc test. (**d**) Representative images of longitudinally split bovine root canals after syringe needle irrigation (SNI), hand file instrumentation (HFI), or HFI + 17% EDTA, illustrating residual Ca(OH)_2_ or AgNPs-100-P-gel. (**e**) Quantitative residual medicament scores based on a four-grade system. Data are expressed as mean ± SD. Statistical comparisons were performed between AgNPs-100-P-gel and Ca(OH)_2_ within the same treatment method: * *p* < 0.05, ** *p* < 0.01, *** *p* < 0.001, and **** *p* < 0.0001, and between AgNPs-100-P-gel in SNI treatment within the various treatment method (& *p* < 0.05) using the Mann–Whitney U test.

A full-wavelength scan of AgNPs-100-P-gel showed a characteristic absorption peak at ~430 nm (Figure 5a). A standard curve from serially diluted 50 μg/mL AgNPs in PBS (OD430) demonstrated strong linearity (R^2^ = 0.9988) (Figure 5b). Measured AgNP concentrations were 10.19–10.73 μg/mL at 1–6 h, 8.80–9.41 μg/mL at 12 h–2 d, and 6.24–7.48 μg/mL at 3–7 d, indicating a gradual decline with a sharp drop after day 3. A residual ~6.24 μg/mL persisted at day 7 (Figure 5c).

### 3.8. Residual Medicament Assessment in Root Canals

In the SNI group, substantial calcium hydroxide remained on canal walls, indicating that conventional irrigation was insufficient (Figure 5d, left). The HFI group showed reduced residues (Figure 5d, middle), with only slight further reduction after a 17% EDTA rinse (Figure 5d, right). In contrast, AgNPs-P-gel was almost completely removed by SNI, HFI, or HFI + EDTA, showing superior removability. Image-based scoring confirmed significantly lower residual scores for AgNPs-P-gel than calcium hydroxide under all conditions (Figure 5e), likely due to its thermoreversible and viscoelastic properties that facilitate removal compared to conventional pastes.

## 4. Discussion

This study evaluated thermoreversible AgNPs-P-gel in terms of gelation temperature, antimicrobial efficacy against planktonic and biofilm bacteria, particle release, cytotoxicity, pro-inflammatory cytokine gene expression, removal efficiency, and SEM findings, providing a comprehensive assessment of its potential as an intracanal medicament. However, endodontic infections are inherently polymicrobial; thus, a mono-species *E. faecalis* model may have limited external validity. In a clinical setting, additional complexities must be considered, including root canal morphology, multispecies microbial interactions, and the host immune response.

Regarding the clinical stability and physical properties of the AgNPs-P-gel, several factors inherent to the intraoral environment must be considered. First, the thermoreversible carrier is engineered with a sol–gel transition temperature optimized to ensure a stable, high-viscosity state at the physiological temperature of 37 °C. The thermoreversible behavior of our system is strongly supported by the established literature. Specifically, Tirnaksiz and Robinson (2005) demonstrated that 20% Pluronic F-127 systems—consistent with our formulation—exhibit a dramatic increase in viscosity from 487.1 Pas at 25 °C to 765.8 Pas at 35 °C, driven by micellar entanglement as temperature rises [60]. Our empirical observations of phase transition are fully consistent with these established rheological profiles. To further validate the clinical utility of this phase change, we evaluated the material’s flow properties according to ISO 6876:2001 standards. At 37 °C, both the water-P-gel and AgNPs-100-P-gel exhibited a flowability of approximately 30 mm, which not only meets the ISO requirement (≥20 mm) but also outperforms the 26–27 mm recorded for the conventional calcium hydroxide-based sealer, Apexit Plus. This confirms that the addition of 100 ppm AgNPs does not compromise the material’s ability to adapt to complex root canal anatomy. While external factors such as cold or hot intake may cause transient intraoral temperature fluctuations, the significant thermal insulation provided by the surrounding enamel and dentin stabilizes the internal root canal environment, preventing unintended phase reversion. Second, although intracanal moisture can theoretically influence polymer concentration, standard clinical endodontic protocols—specifically, the use of paper points to dry the canal—mitigate this risk. The Pluronic-based matrix is designed to maintain its structural integrity and gelation threshold even in the presence of trace residual moisture within the dentinal tubules. Finally, the thermoreversible property offers a distinct advantage concerning flow under apical pressure. By injecting the material in its low-viscosity liquid state at room temperature, it can adapt to intricate root canal morphologies with minimal force. Upon contact with the warm canal walls, rapid gelation occurs, which significantly increases the material’s viscosity and lowers the risk of accidental extrusion beyond the apical foramen compared to conventional pastes that remain at a constant viscosity during placement.

In the immature biofilm model (24 h treatment), AgNPs-50-P-gel showed limited activity against *E. faecalis*, likely from delayed particle release, whereas AgNPs-100-P-gel, calcium hydroxide, and 2% CHX achieved significantly greater bacterial reduction, highlighting the importance of both concentration and release kinetics, consistent with prior dose-dependent AgNP studies [61]. In the mature biofilm model (7 days), all medicaments were highly effective. Notably, even water-P-gel reduced bacterial levels, possibly due to the colloidal matrix creating unfavorable growth conditions—restricted space, prolonged incubation, and nutrient deprivation—independent of antimicrobial action. This supports reports that physical barriers and nutrient stress can impair biofilm viability [62]. These results underscore the need for complementary methods, such as confocal imaging or metabolic assays, to differentiate bactericidal effects from environmental inhibition.

In the present study, 2% CHX was employed as a positive control due to its established status as a potent antimicrobial agent against *E. faecalis* in persistent endodontic infections [63,64]. Although 2% CHX is frequently recommended for its rapid bactericidal effect and high affinity for dental structures, its clinical utility is often hampered by significant cytotoxicity to periapical tissues and a lack of sustained release when used in liquid form. Our results demonstrated that while 2% CHX achieved a high log reduction in *E. faecalis* in both immature and mature biofilms, its performance was statistically matched by the 100 μg/mL AgNPs-Pluronic gel (AgNPs-P-gel). Crucially, the AgNPs-P-gel exhibited a more favorable biocompatibility profile, showing significantly higher cell viability in MTT assays compared to the CHX group (Figure 4). This suggests that the AgNPs-P-gel can achieve the high antimicrobial standard set by 2% CHX while mitigating the risk of tissue irritation. Furthermore, the thermoreversible nature of the Pluronic carrier allows for a more controlled, sustained release of silver ions within the root canal system, addressing the limitations of CHX’s transient activity. Therefore, AgNPs-P-gel represents a promising, safer alternative to 2% CHX for the disinfection of persistent endodontic cases.

The current literature highlights the limitations of traditional medicaments, with Abdullah et al. noting that the antibacterial potency of calcium hydroxide (Ca(OH)_2_ against *E. faecalis* is only significantly enhanced when combined with 2% CHX [65]. Furthermore, Awawdeh et al. argued that brief applications of Ca(OH)_2_ are insufficient, as its efficacy necessitates extended treatment periods [66]. Interestingly, Ca(OH)_2_ demonstrated substantial antimicrobial activity in our study. AgNPs-100-P-gel and calcium hydroxide had similar antibacterial efficacy. We hypothesize this may be a consequence of its suboptimal removability. Our removal efficiency data suggests that standard irrigation fails to fully eradicate Ca(OH)_2_, leading to residual traces that likely skewed the CFU/mL measurements. This was further evidenced by the instant white discoloration of the TSB medium upon the introduction of paper points, confirming the presence of leftover medicament.

In evaluating the clinical potential of AgNPs-P-gel, it is essential to acknowledge the established role of Ca(OH)_2_ as the current gold standard in endodontics, supported by decades of clinical evidence. Our results demonstrated that while Ca(OH)_2_ and 2% CHX are clinically reliable, AgNPs-100-P-gels exhibited significantly greater antimicrobial efficacy against *E. faecalis*. This is particularly relevant as traditional irrigants and dressings often struggle to achieve total bacterial suppression within deep dentinal tubules; for instance, while NaOCl can eradicate surface bacteria, it may fail to completely eliminate *E. faecalis* residing up to 300 µm inside the tubules, where Ca(OH)_2_ also shows limited effects [67]. Furthermore, while the high alkalinity of Ca(OH)_2_ is effective for disinfection, its caustic nature poses risks of dentin brittleness over time and potential periapical tissue damage during accidental extrusion. In contrast, AgNPs-P-gel offers a highly biocompatible alternative with superior handling and simplified removal. Although advanced nanomaterials may incur higher initial costs, the clinical efficiency gained—such as reduced operative time and improved predictability—provides a significant advantage. Beyond infection control, recent evidence highlights that medicaments like iodoform-containing Ca(OH)_2_ can induce apical calcified tissue formation [68]. We hypothesize that while traditional pastes induce mineralization through alkaline-induced necrosis followed by healing, AgNPs-P-gel may promote a more direct regenerative response. By maintaining high cell viability and upregulating wound-healing markers like IL-1β, the gel may serve as a biological scaffold that recruits mineral-producing cells, facilitating the transition from infection control to periapical tissue mineralization.

The efficacy of intracanal medicaments is profoundly challenged by the anatomical complexity of the root canal system, particularly the deep penetration of *E. faecalis* into dentinal tubules. Traditional irrigation with 1.3% NaOCl or dressings with calcium hydroxide often fail to completely suppress *E. faecalis* sequestered within these tubules, despite achieving surface disinfection [67]. This emphasizes the therapeutic problem of bacterial persistence up to 300 μm within the dentin. In our study, the limited antibacterial effect observed in the AgNPs-50-P-gel group, especially in mature biofilms, corroborates the findings that *E. faecalis* is highly resilient in restricted environments. To further understand this resistance, the colonization behavior of *E. faecalis* must be considered. According to the standardized SiO/SiO_2_-microtube in vitro model [69], the diameter of tubular structures strongly influences microbial penetration. While *E. faecalis* can colonize 5.5 μm tubes rapidly, it also successfully invades 2 μm tubes—a diameter similar to human dentinal tubules—over time. Our results show that AgNPs-100-P-gel achieved a reduction of >3 log_10_ CFU/mL, fulfilling the Clinical and Laboratory Standards Institute (CLSI) criteria for antibacterial effectiveness, whereas AgNPs-50-P-gel did not. This suggests that a higher concentration of silver nanoparticles is mandatory to overcome the heavy colonization and reproduction within narrow tubular spaces described in the SiO/SiO_2_ model. Although Ca(OH)_2_ and 2% CHX showed significant inhibitory effects, their clinical use is often hindered by difficult removal and cytotoxicity. The AgNPs-100-P-gel demonstrated no significant difference in antimicrobial efficacy compared to these gold standards in both immature and mature models.

In terms of comparative efficacy, Ting Liu et al. demonstrated that Pluronic gel with 32 µg/mL AgNPs outperformed calcium hydroxide across 1-, 3-, and 9-day intervals against *E. faecalis* biofilms. While that study utilized inductively coupled plasma atomic emission spectroscopy (ICP-AES) to confirm sustained Ag^+^ ion release over 9 days in various physiological buffers [49], our investigation employed spectrophotometry, identifying a distinct release peak around the fifth day. Despite these methodological differences in release kinetics, both studies affirm that the thermoreversible AgNP-gel provides a sustained drug profile essential for robust antimicrobial action. Differences in gel composition, particle size, release kinetics, bacterial strain, and experimental conditions may explain this variation [70]. To further clarify these release dynamics and their biological impact, future investigations should incorporate advanced electrochemical and chemical analysis techniques to achieve a more granular understanding of the delivery system’s potential. Furthermore, the artificial SiO/SiO_2_-microtubes model allows for precise control over pore diameters and can effectively simulate microbial infiltration through dentinal tubules. This represents a promising experimental framework for further investigation in future studies [69].

The clinical viability of any intracanal medicament is fundamentally predicated on its safety profile, specifically its ability to exert antimicrobial effects without inducing significant localized tissue damage. In the present study, we conducted a rigorous evaluation of the cytotoxic potential of several endodontic agents using the MTT metabolic assay, employing a diverse range of extraction ratios and exposure durations to simulate various clinical scenarios. Our quantitative analysis revealed a distinct and consistent gradient of biocompatibility among the tested groups. Specifically, 2% CHX exhibited the most pronounced inhibitory effect on cell viability, suggesting a high level of cytotoxicity that may raise concerns during prolonged clinical contact. Conversely, the AgNPs-P-gel demonstrated significantly higher cell survival rates compared to the other groups. This superior biocompatibility was followed by calcium hydroxide, with 2% CHX consistently ranking as the most cytotoxic agent. By maintaining high cell viability across different experimental models, the AgNPs-P-gel distinguishes itself as a safer, more cytocompatible alternative to traditional medicaments.

In healthy mice, systemic injection of 2 mg/kg AgNPs has been shown to upregulate inflammatory genes—including *TNF-α*, *CXCL1*, *TGF-β*, *HO-1*, and *IL-4*—within the spleen [71], yet AgNPs administered via intravenous catheterization can effectively alleviate experimental colitis, with an anti-inflammatory efficacy that depends on nanoparticle shape and diameter [72]. Our study observed that while all tested medicaments, including water-P-gel, Ca(OH)_2_, and 2% CHX, induced inflammatory gene expression in L929 cells—consistent with prior reports of Ca(OH)_2_ and 2% CHX stimulating cytokines in fibroblasts and odontoblast-like cells [73,74]—AgNPs-P-gel demonstrated superior cell viability at both 24 and 72 h. Notably, the AgNPs-50-P-gel group exhibited a recovery in cellular activity by 72 h, suggesting a favorable environment for recovery compared to the sustained toxicity of Ca(OH)_2_ and 2% CHX. Regarding the inflammatory profile, while all groups upregulated *IL-6* and *TNF-α*, AgNPs-P-gel specifically influenced *IL-1β* expression; although these are traditionally classified as pro-inflammatory markers, they are critical mediators in the initial stages of wound healing and tissue signaling. Therefore, we hypothesize that AgNPs-P-gel may support a regenerative healing response rather than merely providing low cytotoxicity. Compared to water-P-gel, AgNPs-P-gel showed a slightly higher trend in cell viability and a lower inflammatory response, though the differences were not significant (Figure 4). While the systemic inflammation risk from intracanal use is likely minimal, future work should utilize RT-qPCR or ELISA to monitor inflammatory markers in Pluronic-based delivery systems and evaluate diverse cell types, such as immune cells, dental pulp stem cells, and osteoblasts, to fully elucidate the potential of AgNPs-P-gel in promoting mineralized barriers or tissue regeneration.

Several limitations of this study should be acknowledged. First, the use of a mono-species biofilm model lacks the polymicrobial complexity of clinical endodontic infections [75]. Second, while over-instrumented bovine canals provided a standardized environment, their differences from human teeth in anatomy, mineral content, and tubule density may affect generalizability [76]. Methodologically, the gelation assessment was primarily qualitative, and potential optical interference in OD600 readings suggests that supplementary viability assays would be beneficial. Furthermore, this preliminary investigation lacked full nanoparticle characterization (e.g., surface charge and long-term stability). Finally, the small sample size limited statistical power; therefore, larger studies incorporating multispecies or patient-derived models and quantitative rheological analysis are essential to further validate the clinical potential of this approach.

## 5. Conclusions

In summary, within the limitations of this in vitro and ex vivo study, the AgNPs-P-gel demonstrated antibacterial efficacy against *E. faecalis* and *S. mutans* comparable to calcium hydroxide and 2% CHX, while exhibiting significantly lower cytotoxicity and superior removability. These findings suggest that its thermoreversible nature and biocompatibility may offer a potential alternative for root canal disinfection. However, further in vivo research and clinical trials are necessary to evaluate the long-term biological effects and clinical predictability of this formulation in the complex environment of the human oral cavity.

## Figures and Tables

**Table 1:** List of pro-inflammatory cytokine genes and primer sequences.

Genes	Forward Primer	Reverse Primer
*GAPDH*	TATGTCGTGGAGTCTACTGGT	GAGTTGTCATATTTCTCGTGG
*IL-1β*	TGGACCTTCCAGGATGAGGACA	GTTCATCTCGGAGCCTGTAGTG
*TNF-α*	GGTGCCTATGTCTCAGCCTCTT	GCCATAGAACTGATGAGAGGGAG
*IL-6*	TGTACTCCAGGTAGCTATGG	GTTCTCTGGGAAATCGTGGA

## Data Availability

The original contributions presented in this study are included in the article. Further inquiries can be directed to the corresponding authors.

## Abbreviations

The following abbreviations are used in this manuscript:

AgNPs-P-gel	thermoreversible silver nanoparticle (AgNP)-loaded Pluronic gel
MIC	minimum inhibitory concentration
MBC	minimum bactericidal concentration
SEM	scanning electron microscope
CHX	chlorhexidine
*IL-6*	*interleukin-6*
*IL-1β*	*interleukin-1β*
*TNF-α*	*tumor necrosis factor-α*
*GAPDH*	*glyceraldehyde 3-phosphate dehydrogenase*
SNI	standard needle irrigation
HFI	hand file irrigation
